# Setting Process Monitoring of Cement Paste Using Electromechanical Impedance of Piezoelectric Patch

**DOI:** 10.3390/ma15228114

**Published:** 2022-11-16

**Authors:** Jun-Cheol Lee, Chang-Yong Yi

**Affiliations:** 1 Department of Architecture, Seowon University, Cheongju 28674, Republic of Korea; 2 Intelligent Construction Automation Center, Kyungpook National University, Daegu 41566, Republic of Korea

**Keywords:** electromechanical impedance, piezoelectricity, cement paste, setting

## Abstract

Electromechanical impedance (EMI) sensing is typically applied to monitor the setting of fresh cement paste. In this study, an experimental test is conducted to demonstrate the effectiveness of EMI sensing for monitoring the setting time of fresh cement paste. A square piezoelectric (PZT) patch was embedded in fresh cement paste and the EMI of PZT patch was continuously monitored for 12 h. The results demonstrate that EMI sensing provides significant signals during the first 12 h of the cement-setting process.

## 1. Introduction

The setting of cement-based materials, such as concrete, presents a transition phase between fluid and rigid states [1]. In this process, the material ceases to behave as a liquid and begins to exhibit the characteristics of a solid material. Knowledge regarding the setting process of cement-based materials is crucial, as it can be used to regulate the times of mixing, transit, and placing, to gauge the effectiveness of various set-controlling admixtures, and to plan the scheduling of finishing and form-removal operations. The setting process involves two setting times, i.e., the initial and final setting times [1]. At the initial setting time, the paste begins to stiffen considerably and can no longer be molded; at the final setting time, the cement has hardened to a level that allows it to sustain some loads.

Standard test methods are available for practically determining these two important times in the setting process of cement-based materials at the laboratory scale [2,3]. For example, in the test method designated in ASTM C191 (Vicat needle penetration test), the initial setting time is defined as the time at which the needle penetrates 25 mm into the cement paste sample, and the final setting time is the time at which the needle does not sink visibly into the sample [2]. Although standard test methods provide practical values for determining the initial and final setting times, these values may not correspond to the exact initial and final setting times, as they are fixed regardless of the composition of the cement-based materials. 

Generally, the initial setting occurs within 2–4 h, and the final setting occurs 5–8 h after mixing. The initial and final setting times of cement-based materials vary with the composition of the materials, additives, and environmental conditions (e.g., temperature and humidity) [1]. Because the setting occurs during the consecutive microstructure formation of hydrating cement-based materials, which begins immediately after mixing, continuous monitoring beginning at the mixing stage is required to capture the exact initial and final setting times and to observe the evolution of the fundamental physical properties in the subsequent hydration process. In this regard, researchers have focused on developing techniques that allow one to continuously monitor the evolution of the mechanical properties of hydrating cement-based materials [4,5,6,7,8,9,10,11,12].

Recently, piezoelectric materials have been used in nondestructive monitoring techniques in the construction industry. In particular, electromechanical impedance (EMI) sensing techniques, utilizing piezoelectric ceramic (PZT) wafers, have demonstrated significant potential for the in situ monitoring of early age concrete [13,14,15]. The basic principle of EMI sensing is simple. A PZT wafer coupled (by bonding or embedding) with a monitored (host) structure driven with a sinusoidal voltage causes the coupled area of the structure to vibrate, due to the direct effect of the piezoelectric material. Additionally, this vibration response causes an electrical response—typically an electric current—in the PZT transducer, due to the converse piezoelectric effect. Liang et al. [16] proposed a one-dimensional electromechanical interaction model for a PZT wafer surface bonded to a host structure, as follows:

(1) $$Y\left(\omega \right)=j\omega \frac{w_{A\;}l_{A}}{h_{A}}\left[\frac{d_{31}^{2}{\bar{Y}}_{11}^{E}Z_{A}}{Z_{A}+Z}\frac{{\tan(\mathrm{kl}}_{A})}{{\mathrm{kl}}_{A}}+{\bar{\varepsilon }}_{33}^{T}-d_{31}^{2}{\bar{Y}}_{11}^{E}\right],$$

where $Y\left(\omega \right)$ is the electrical admittance (inverse of impedance) of a PZT patch bonded to a host structure, j = $\sqrt{-1}$, ω the excitation frequency, $w_{A\;}$ is the width of the PZT patch, $l_{A}$ is the length of the PZT patch, $h_{A}$ is the thickness of the PZT patch, Z is the host structural impedance, $Z_{A}$ is the impedance of the PZT patch, k is the wave number, ${\bar{\varepsilon }}_{33}^{T}$ is the dielectric constant of the PZT patch in a 3–3 direction, $d_{31}$ is the piezoelectric constant, and ${\bar{Y}}_{11}^{T}$ is the complex Young’s modulus of the PZT patch. As the mechanical impedance of the PZT wafer does not change over the monitoring period, the equation indicates that a change in the mechanical impedance of the host structure directly results in a change in the electrical impedance measured by the transducer. Therefore, without loss of generality, the evolution of the mechanical impedance of hydrating cement-based materials can be monitored by continuously tracking the electrical impedance of the PZT patch coupled with fresh cement-based materials. In this study, an EMI sensing technique utilizing a PZT wafer was applied to monitor the setting process of cement-based materials.

In most monitoring applications that adopt EMI sensing, a PZT wafer is adhesively bonded to the surface of the host structure to couple the patch and the structure [13,14,16]. However, surface bonding is not suitable for monitoring the setting of cement-based materials using PZT wafers because the state of cement-based materials during the setting process is almost liquid. For this reason, techniques for measuring EMI by embedding a PZT sensor directly into a cementitious material have been introduced [17,18,19,20,21]. The embedding of a PZT patch provides two important technical advantages. First, the system can be monitored once the paste is mixed. Second, the bonding condition between the wafer and host structure, which significantly affects EMI measurements in a surface-bonded approach [13] and is typically assumed to exhibit a constant value, need not be considered, because the bonding can be achieved organically via the hydration process. In other words, the bonding condition is an indicator of the mechanical impedance evolution of the hydrating cement-based material. A technical concern pertaining to the embedment of PZT wafers is the short-circuit condition when the PZT wafer is embedded in a “liquid state” material, e.g., the fresh cement paste in this study. To prevent this short-circuit condition, a thin coating of unsaturated polyester resin is applied on the surface of the PZT wafer before its embedment.

## 2. Materials and Methods

**Figure 1:**
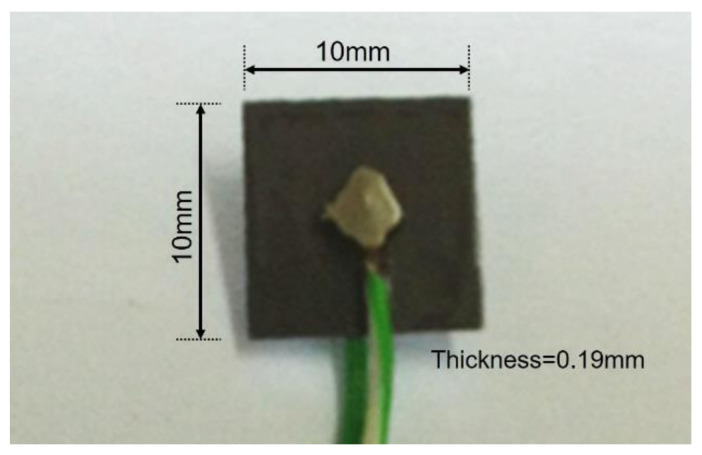
PZT patch used in this study.

**Figure 2:**
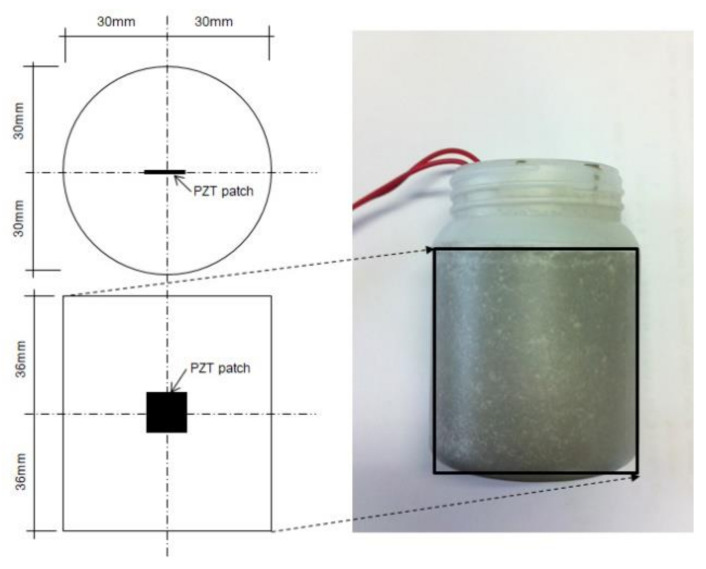
Cement paste sample and embedding position of PZT patch.

In this study, a square PZT patch (Piezo.com, T107-H4NO-2929) measuring 10 mm × 10 mm × 0.19 mm was used as an EMI sensor. An unsaturated polyester resin was thinly coated on the surface of the PZT patch to prevent short-circuit conditions. Figure 1 shows the PZT patches used in this study. Ordinary Type I Portland cement and distilled water were used to prepare cement paste specimens. Distilled water was used as the mixing water to prevent adverse effects on cement hydration. A cement paste with a water-to-cement ratio of 0.4 was prepared in accordance with ASTM C 305 [22]. The cement paste was cast in a disposable mold (with a diameter of 60 mm and a height of 72 mm) immediately after mixing, and the PZT patch was embedded around the center of the mold. Immediately after casting, the electromechanical admittance (inverse of impedance) of the PZT embedded in the cement paste was measured. Figure 2 shows the embedding position of the PZT patch.

**Figure 3:**
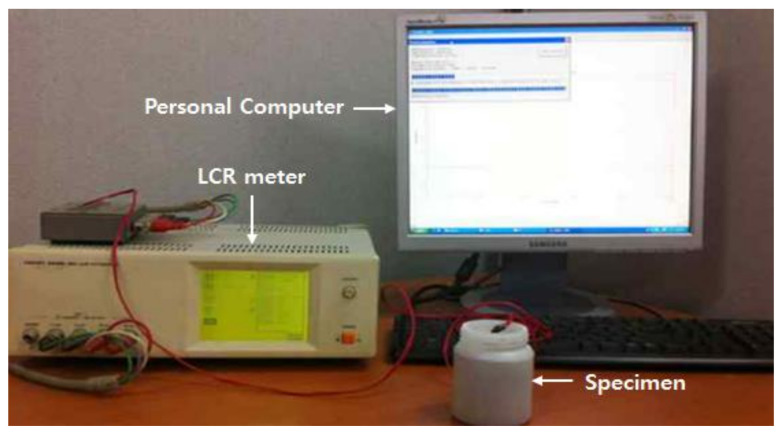
Experimental setup for EMI-based monitoring of cement paste setting.

A commercial LCR (Inductance (L), Capacitance (C), and Resistance (R)) meter (HIOKI, 3532-50 LCR HiTESTER) was used for admittance monitoring as a function of material age. The admittance of the PZT embedded in the cement paste was measured every 5 min for 12 h (144 time steps). At each measurement time step, the admittance signature (frequency range: 20–480 kHz with 500 Hz intervals) was measured five times repeatedly and averaged to remove incoherent noise. All the measured data were recorded using a personal computer via a GP-IB interface attached to the LCR meter. The experimental setup for the embedded PZT impedance measurements is shown in Figure 3.

## 3. Results and Discussions

### 3.1. Admittance of PZT

**Figure 4:**
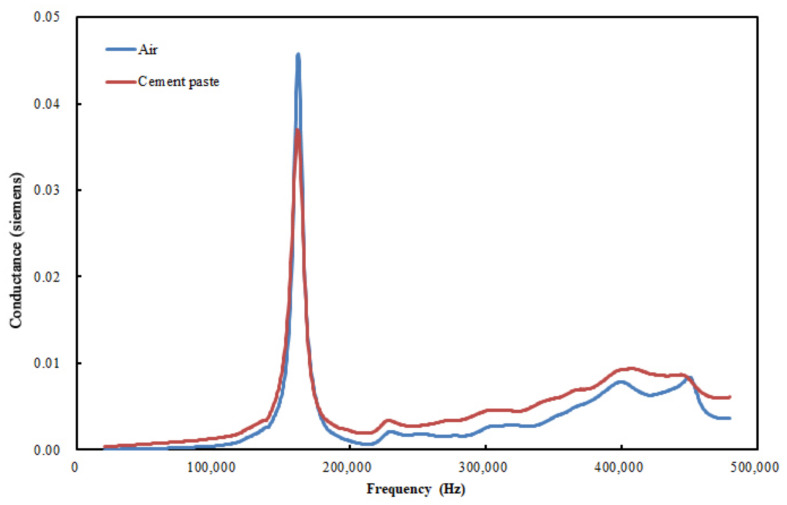
Conductance (real component of admittance) of PZT patch in air and in freshly mixed cement paste.

Figure 4 shows the real component of the admittance (conductance) of the PZT in air (i.e., before embedment) and in a freshly mixed cement paste. As shown, the peak locations for both cases are the same (165 kHz), but the magnitude of the peak for the case involving cement paste is lower than that for the case involving air. In addition to the peak location, the conductance in the paste is higher than that in air. These signal changes are due to the mechanical impedance change in the surrounding material of the PZT. This suggests that the mechanical impedance of fresh cement paste, Z, as expressed in Equation (1), is higher than the mechanical impedance of air, thus verifying that the cement paste is initially in the liquid state.

**Figure 5:**
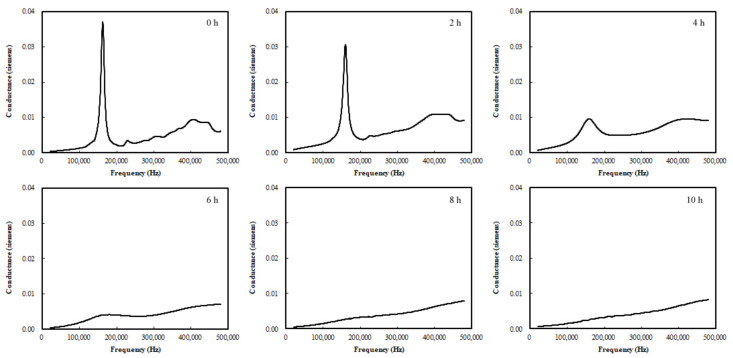
Conductance (real component of admittance) of PZT patch in cement paste at different material ages.

Figure 5 shows the conductance of the PZT patch in the cement paste at different ages. The results show that the signal changed significantly as the material aged. The first mode resonance at the initial age occurred at approximately 160 kHz, where a sharp and high peak magnitude of 0.037 S was recorded. As the cement paste aged, the magnitude of the peak decreased, and the shape of the peak became blunter and unrecognizable after 6 h.

The magnitude decrease in the resonance peak can be explained using Equation (1). Based on this equation, the magnitude of admittance decreases with an increase in the mechanical impedance of cement paste. Because the electromechanical properties of PZT materials are age-independent, the signal decrease is due to the increase in the mechanical impedance of the cement paste; this suggests that the proposed method can be used for monitoring the stiffness gain (impedance increase) of the cement paste caused by cement hydration. However, the blunt shape of the resonance peak suggests that the damping of the cement paste increases with age. Notably, the fresh cement paste is neither liquid nor solid; hence, both its stiffness and damping properties change as it hydrates.

Additionally, based on Figure 5, the magnitude of the admittance at ~400 kHz, which is approximately the second resonance frequency, indicates a slight signal change from 1 to 3 h and an abrupt signal change between 3 and 4 h. Subsequently, the signal remained primarily unchanged. Compared with the first resonance peak behavior, the second resonance peak behavior is less evident; hence, using the first resonance peak may provide more reliable results for monitoring.

### 3.2. Metrics for Evaluating PZT Admittance Change

To evaluate the conductance change as a function of the material age, we utilized three different metrics: (1) the relative peak (RP) magnitude of the first resonance, (2) the relative area (RA) under the first resonance with a bandwidth of 60 kHz, and (3) the root-mean-square deviation (RMSD) of the first resonance with a bandwidth of 60 kHz. The bandwidth of 60 kHz included the first resonant frequency of the conductance (165 kHz); hence, the frequency range of conductance evaluated for the metric ranged from 135 to 195 kHz. Each metric is expressed mathematically as follows:

(2) $$\mathrm{RP}\left(t\right)=\frac{G_{t,p}}{G_{0,p}}$$

(3) $$\mathrm{RA}\left(t\right)=\frac{\sum_{i=1}^{n}G_{t,i}}{\sum_{i=1}^{n}G_{0,i}}$$

(4) $$\mathrm{RMSD}\left(t\right)=\overset{n}{\underset{i=1}{\sum }}\sqrt{\frac{{\left(G_{t,i}{-G}_{0,i}\right)}^{2}}{{\left(G_{0,i}\right)}^{2}}}$$

where $G_{t,p}$ is the conductance at the peak frequency at age t, $G_{0,p}$ is the conductance at the peak frequency at the initial age, $G_{t,i}$ is the conductance at the i-th location of the bandwidth at age t, and $G_{0,i}$ is the conductance at the i-th location of the bandwidth at the initial age. The initial age represents the time required to mix water and cement paste.

The RP and RA share the same conceptual meaning, i.e., the ratio of the peak magnitude at one age to that at the initial age. The RA is introduced, as monitoring the peak location of conductance beyond a certain material age is difficult. Meanwhile, RMSD, which is a widely used metric in structural health monitoring studies, refers to the signal difference between conductance at one age and that at the initial age.

**Figure 6:**
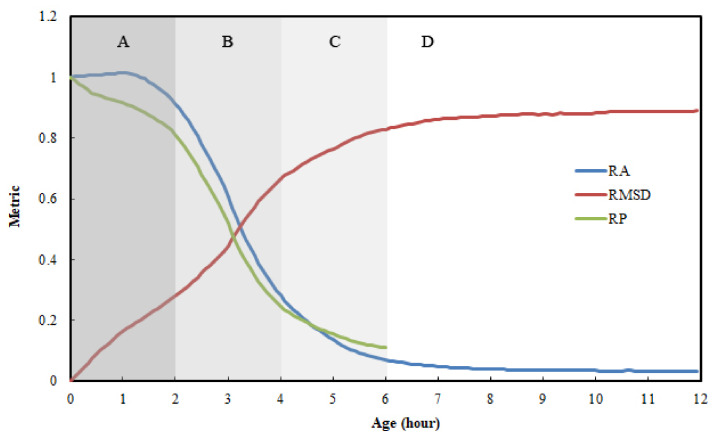
Metric as a function of material age.

Figure 6 shows the three metrics as a function of the material age. The RP is indicated only until the age of 6 h because it becomes more difficult to monitor the peak after the age of 6 h; this is because beyond 6 h, the peak becomes blunter, which complicates the detection of the peak. The metric RP decreases linearly until 20 min; subsequently, it decreases gradually until 2 h, decreases rapidly between 2 and 4 h, approaches the steady state between 4 and 6 h, and maintains at a steady state after 6 h. The metric RA increases slight until 1 h, decreases rapidly between 2 and 4 h, approaches a steady state, and maintains at a steady state after 6 h. The RMSD shows a similar trend, but it increases from zero to a steady-state value of 0.88. Based on these observations, the curve trends can be described based on four regions in the time domain, as follows:

(A) 0–2 h—gradual changes;
(B) 2–4 h—rapid changes;
(C) 4–6 h—approaching the steady state;
(D) beyond 6 h—remains in the steady state.

Typically, the initial setting occurs within 2–4 h, and the final setting occurs 5–8 h after the cement paste is mixed [3]. Based on this general observation, one can infer that the initial setting time occurs in Region B (rapid change in the curves), and the final setting time occurs in Region D (steady state). This suggests a strong physical relationship between the setting process and PZT impedance. The initial setting represents the transitional state from a liquid-like material to a solid-like material; hence, one may expect a rapid change in the PZT signal. Meanwhile, the final setting represents an evolution from a transitional state to the solid state; hence, one may expect the PZT signal to converge to a certain value.

## 4. Conclusions

The EMI of an embedded PZT patch in cement paste provides a distinctive signal close to the setting times. The change in PZT impedance corresponding to the initial setting regions was reflected as a rapid change in the signal, whereas that corresponding to the final setting region was indicated by the convergence of the signal to a certain value. For monitoring the setting time, the PZT patch detected time signals that were related to the initial and final setting of the cement pastes.

The two primary advantages of the embedded PZT method for monitoring cement setting are as follows: (1) It can yield a nondestructive continuous signal change, in which the degree of the setting process is reflected; and (2) It allows the setting process to be monitored once the cement paste is mixed via the embedment of a PZT patch in the cement paste.

## Data Availability

Not applicable.
